# Telomerase activity, estrogen receptors (α, β), Bcl-2 expression in human breast cancer and treatment response

**DOI:** 10.1186/1471-2407-6-206

**Published:** 2006-08-15

**Authors:** Blanca Murillo-Ortiz, Horacio Astudillo-De la Vega, Sebastian Castillo-Medina, JM Malacara, Luis Benitez-Bribiesca

**Affiliations:** 1 Unidad de Investigacion Clinica, UMAE no. 1 Bajio, IMSS Leon Guanajuato, Mexico; 2 Unidad de Investigacion Medica en Enfermedades Oncologicas, Centro Medico Nacional Siglo XXI, IMSS, CP 06720, DF, México; 3 Instituto de Investigaciones Medicas, Universidad de Guanajuato, Mexico

## Abstract

**Background:**

The mechanism for maintaining telomere integrity is controlled by telomerase, a ribonucleoprotein enzyme that specifically restores telomere sequences, lost during replication by means of an intrinsic RNA component as a template for polymerization. Among the telomerase subunits, hTERT (human telomerase reverse transcriptase) is expressed concomitantly with the activation of telomerase. The role of estrogens and their receptors in the transcriptional regulation of hTERT has been demonstrated. The current study determines the possible association between telomerase activity, the expression of both molecular forms of estrogen receptor (ERα and ERβ) and the protein bcl-2, and their relative associations with clinical parameters.

**Methods:**

Tissue samples from 44 patients with breast cancer were used to assess telomerase activity using the TRAP method and the expression of ERα, ERβ and bcl-2 by means of immunocytochemical techniques.

**Results:**

Telomerase activity was detected in 59% of the 44 breast tumors examined. Telomerase activity ranged from 0 to 49.93 units of total product generated (TPG). A correlation was found between telomerase activity and differentiation grade (p = 0.03). The only significant independent marker of response to treatment was clinical stage. We found differences between the frequency of expression of ERα (88%) and ERβ (36%) (p = 0.007); bcl-2 was expressed in 79.5% of invasive breast carcinomas. We also found a significant correlation between low levels of telomerase activity and a lack of ERβ expression (p = 0.03).

**Conclusion:**

Lower telomerase activity was found among tumors that did not express estrogen receptor beta. This is the first published study demonstrating that the absence of expression of ERβ is associated with low levels of telomerase activity.

## Background

The mechanism for maintaining telomere integrity is controlled by telomerase, a ribonucleoprotein enzyme that specifically restores telomere sequences lost during replication by means of an intrinsic RNA component as a template for polymerization. Telomerase has 2 core functional components that are required for its activity the catalytic subunit of human telomerase reverse transcriptase (hTERT), and a telomerase RNA template (hTR). It is activated in most immortal cell lines in culture and in most malignant tumors [1] and is detected in 85–90% of human cancer specimens [2]. Telomerase activity is found in most tumor types including colorectal, breast, prostate, ovarian carcinomas and others. It also is increased in pre-invasive lesions of the breast, such as ductal carcinoma *in situ *(DCIS), suggesting that telomerase activity is activated early in breast carcinogenesis [3]. Among the telomerase subunits, TERT is concomitantly expressed with the activation of telomerase during cellular immortalization and tumor progression [4], but the pattern and level of hTERT expression are heterogeneous, with ~75% of tumors expressing bulk hTERT mRNA levels equal to or less than those observed in normal breast tissue; moreover, tumors expressing higher than normal levels of hTERT mRNA are more likely to be histopathologic grade 3, with a high fraction of cells in S-phase [5].

Pre-invasive breast cancer may exhibit overexpression of c-erbB-2 and bcl-2 oncoproteins, mutations in p53, and alterations in cell cycle regulators [6,7]. Bcl-2 is a proto-oncogene that codes for an inner mitochondrial membrane protein [8]. The Bcl-2 gene originally was described in follicular B-cell lymphoma, in which t (14;18) chromosomal translocation results in a high level of Bcl-2 gene expression [9]. Bcl-2 overexpression in human breast cancer is reported to be associated with the presence of estrogen receptors (ER), small tumor size, low tumor grade, and a favorable prognosis [10-17], despite contradictory results [18,19]. Misiti *et al *[20] proved the role of estrogens and their receptors in the transcriptional regulation of hTERT. Kyo *et al *[21] reported the upregulation of telomerase activity in estrogen receptor (ER)-positive MCF-7 cells treated with 17-beta-estradiol [21]. Tkalogeraki *et al *[22] reported telomerase activity in 72% of breast carcinomas, and an association was identified between telomerase activity, estrogen receptors and HER-2 expression, and histological grade and lymph node status.

We conducted the present study to determine whether an association between telomerase activity and the expression of both molecular forms of estrogen receptor (ERα and ERβ) and the protein bcl-2 exists. We also investigated if telomerase function is predictive of response to chemotherapeutic drugs, and if it is associated with any clinical parameters in a group of patients with infiltrating ductal breast carcinoma.

## Methods

### Patients and treatment

Tumor samples were collected from breast carcinoma patients in the Department of Oncology, and from the Rodolfo Padilla Padilla Foundation and the National Cancer Institute of Mexico. Informed consent for the experimental use of samples was obtained from all patients. Forty-four breast tumor tissues were obtained using tru-cut biopsies. Additionally, normal human breast tissue specimens were obtained from 2 women (aged 45 and 49) at the time of reductive mammoplasty. Each specimen was sectioned immediately after surgery into two portions; one was snap frozen for the telomerase (TRAP) assay; and the other was fixed in 10% formalin and embedded in paraffin for IHC, as described below. Patients were treated with 6 cycles of FEC-based chemotherapy with 5-fluorouracil (500 to 600 mg/m^2^), epirubicin (60 mg/m^2^), and cyclophosphamide (600 mg/m^2^). Surgery consisted of modified radical mastectomy, which was followed by radiotherapy.

### Tumor response criteria

After chemotherapy, all patients were evaluated for response by physical examination, chest radiography, hepatic ultrasound, and computerized tomography scan. Response to chemotherapy was classified either as 1) complete clinical response (CR), defined as total disappearance of tumor; 2) partial response (PR), defined as >50% reduction in tumor size; 3) no-response (NR), defined as <50% reduction in tumor size, but no clear progression; or 4) progressive disease (PD), defined as >25% increase in the size of existing lesions or the appearance of new tumor lesions [23].

### Preparation of tissue exctract

Minced tissue samples were suspended in IX CHAPS Lysis buffer and homogenized with a motorized pestle for 20 seconds and incubated at ice temperature for 30 min. Samples were spined at 12000 × g for 20 min at 4°C. Aliquotes of the supernatant were used for telomerase determination.

### TRAP assay

Telomerase activity was measured using the TRAP method (*TRAPEZE^® ^XL *telomerase detection kit, Invitrogen, Manhattanville, NY), in accordance with the method proposed by Kim *et al *[25]. The supernatant was poured into microtubes, frozen in liquid nitrogen, and stored at -80°C. Extracts containing 1.5–2.0 μg/μl protein were used for telomerase assays.

### Standard curve

A standard curve was generated, based upon measurement of the reactions performed with a TSR8 (control template), in order to calculate the amount of TS primers with telomeric repeats extended by telomerase in each tumor extract. Serial dilutions (1:5) of the stock concentration (0.2 amoles/μL) were prepared to obtain TSR8 concentrations of 0.04 amoles/μL, 0.008 amoles/μL, and 0.0016 amoles/μL.

### Telomerase positive control and heat inactivation control

In order to validate the analysis, a HeLa cell extract from the 1000 cells provided with the kit was prepared during each series of reactions. Each aliquot of lysate was diluted with CHAPS XL Buffer before use. Considering that telomerase is a heat-sensitive enzyme, a negative control for every tumor sample was tested. We heated 10 μL of each sample at 85°C for 10 min prior to *TRAPEZE^® ^XL *Kit assay performance.

### Quantization of telomerase activity

The reaction products (50 μL) of the TRAP assay were mixed with 150 μL of Buffer (10 mM Tris-HCl, pH 7.4, 0.15 M NaCl, 2 mM MgCl_2_). They were transferred to a black 96-well plate to obtain maximum performance. We used a compatible Spectrofluorophotometer plate reader (Fluoroscan, Labsystem, Finland) to quantify the fluorescence value generated. A standard curve using the TSR8 template was used to validate quantitative results.

### Histopathology and immunohistochemistry

Representative tissues were fixed in buffered formalin and paraffin-embedded. Sections were cut at 5–6 μm and placed on slides previously covered with DL-lysine. One slide was stained with hematoxilin-eosin for analysis and classification by 2 independent pathologists, using a 3-tiered nuclear and histologic grading system, in accordance with the Bloom and Richardson grading system. The other unstained sections were de-paraffinized and used for immunohistochemistry. Epitopes were retrieved by autoclaving in 10 mM citric acid buffer, pH 6.1 for 15 min. Slides were incubated overnight with the following primary antibodies: ΔC21 anti-human polyclonal Bcl-2; (Santa Cruz Biotechnology, 1:100), F-10 anti-human monoclonal ERα (Santa Cruz Biotechnology, 1:100) and H-150 anti-human polyclonal ERβ (Santa Cruz Biotechnology, 1:100), all in 1% bovine serum albumin-phosphate buffered saline (BSA-PBS). These antibodies against ERs have been successfully used to distinguish the two molecular forms in experimental breast cancer [26]. Immunostaining was carried out using the Universal DAKO LSAB2 system according to manufacturer's instructions. Alternate sections were processed omitting the primary antibody, and using instead commercial mouse IgG cocktail (N-Universal Negative Control, DAKO N-1698 Carpinteria, CA) and rabbit IgG cocktail (N-Universal Negative Control, DAKO N-1699 Carpinteria, CA) to generate negative controls. Immunoreactivity was estimated in100 malignant cells to calculate the percentage positive cells in each set. Additionally, normal breast tissue samples obtained at the time of reductive mammoplasty were used as normal controls.

### Statistical analysis

Fisher's exact test was used to determine the association between telomerase activity and tumor size, tumor grade, nodal status and Bcl-2 expression, using *p *<*0.05 *as level of significance. Correlations were tested by means of Spearman rank correlation coefficients. The relative expressions of estrogen receptors were analyzed using paired t-tests. For regression analysis, the dependent variable was telomerase activity, again using p < 0.05 as level of significance.

## Results

### Patient characteristics and clinical response

Patient characteristics and tumor response are shown in table 1. Patients had a median age of 52 years (range 27–77 years). At the end of chemotherapy, complete response (CR) was seen in 29 of 44 (65.9%); 15 (34.1%) had either a partial response (PR) or no response (NR).

### Telomerase activity in human breast carcinoma tissues

Telomerase activity was detected in 26 (59%) of the 44 breast examined tumors, and it was negative in the remaining 18 (41%) samples. TRAP assay revealed that, among the 44 breast carcinomas, the range of telomerase activity varied greatly, ranging from 0.0 to 49.93 (Table 2). The mean telomerase activity was 9.94 ± 18.05 TPG units in breast cancer and none in normal tissue. We examined the possible association between this enzyme activity and the clinical stage of breast carcinoma. An association was identified between telomerase activity and higher histologic grade (p = 0.03; Table 3). We found no significant correlation between telomerase activity and tumor stage. No correlation was observed between telomerase activity and disease course, age, or menopausal status.

### Telomerase activity and response to treatment

The association between telomerase activity and response to treatment, as a dependent variable, was also evaluated by means of logistic regression analysis, to take into consideration the effects of the other covariates investigated, including age, cancer stage, histologic grade, status of estrogen receptors alpha and beta, and bcl-2. Only clinical stage reached statistical significance as an independent marker of response to treatment, but telomerase activity had no predictive value.

### Immunohistochemistry

**Figure 1:**
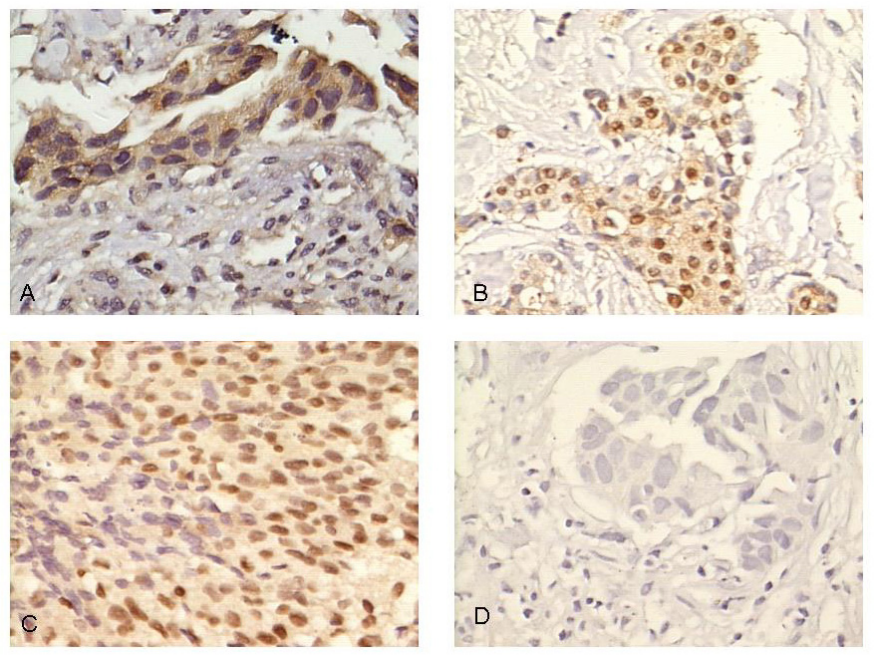
In this panel we show sections of invasive ductal carcinoma of the breast incubated with different primary antibodies and stained with the streptavidin-biotin-peroxidase complex. A) Bcl-2 antibody is localized in the cytoplasm of malignant cells and infiltrating lymphocytes are also immunoreactive. B) ERα antibody is present primarily in the nuclei of malignant cells; some cells are negative. C) ERβ antibody is localized in the nuclei of most cancer cells. D) Negative control.(400×).

Positive staining for estrogen receptors alpha and beta were primarily found in the nuclei of breast cancer malignant cells. Some immunostaining was also seen in the cytoplasm of the same cells. Neighboring cells such as fibroblasts, macrophages, lymphocytes and endothelial cells, were unstained. The bcl-2 immunostaining was found in the cytoplasm of cancer cells. Infiltrating lymphocytes showed also positive bcl-2 staining (Figure 1). Normal breast tissue showed scattered staining in the duct epithelium for estrogen receptors alpha and only occasional nuclear staining for estrogen receptors beta. The percentage of immunoreactivity of the ERα (54 ± 41.7%), ERβ (28.5 ± 41%) and bcl-2 (60.8 ± 38.2%) in 44 invasive ductal carcinomas are shown in Table 2. Differences were found between the expression of ERα 39 (88%) and ERβ 16 (36%) (p = 0.007); the expression of bcl-2 was seen in 35 (79.5%) invasive breast carcinomas (Table 1). There was a higher frequency of bcl-2 expression in stage II as compared with Stage III/IV (p = 0.02; Table 4). No relationship was observed between bcl-2 and node status, tumor size, or histological grade. However, a strong positive relationship was observed between bcl-2 and ERα, with 26 of 35 (74.2%) bcl-2 positive tumors also being ERα positive (p = 0.02). The expression of bcl-2 did not correlate with ERβ.

### Association between telomerase activity and expression of ERβ and ERα

Comparing the groups with and without ERβ expression, the latter had lower telomerase activity (p = 0.03). In contrast, telomerase activity was not related to ERα expression (p = 0.61). These overall findings indicate that ERβ is associated with low telomerase activity.

## Discussion

Our results showed that 59% of ductal breast carcinomas had some telomerase activity confirming previous studies in neuroblastomas, [27] lung carcinomas, hepatocellular cancers [28] gastrointestinal cancers [29] and breast cancers [27]. It is known that activation of telomerase by tumor cells can ensure survival with genomic instability. Therefore, although telomerase might not directly act on the carcinogenetic process it certainly is a promoter [30]. The association of telomerase activity with higher expression of Bcl-2 although not statistically significant in our study, highlights the fact that this antiapoptotic protein might add another factor that promotes carcinogenesis. We also found that the two molecular forms of ER are expressed differently. ERα was found in 88% of cases while ERβ was seen only in 36% of our breast cancers.

ERα and ERβ have some overlapping tissue distribution, but also display high relative tissue-specific expression [14-17]. Using immunochemical analyses, Roger et *al *[31] found a higher percentage of ERβ positive cells in normal mammary glands than in non-proliferative benign breast disease (85%), proliferative disease without atypia (18.5%) and carcinoma *in situ *(33.8%). In contrast, an increase in ERα protein expression was noted during cancer progression. Large studies of ERβ in breast cancer show that its expression does not correlate with absence of ERα (-), but that some tumors and cell lines express both [32]. It has been established that ERα mRNA is increased in breast cancer, while in normal mammary tissue the expression of ERα mRNA is low or absent [32,33]. Others have associated the presence of the mRNA of ERβ with poor prognosis, positive lymph nodes, and tamoxifen resistance [34]. We identified differences in the frequency of expression of the estrogen receptors; beta receptor expression was positive only in 36% of the cases, versus 88% for the alpha receptor, a percentage similar to that reported by Shaw [35].

In chemically induced mammary cancers in rats Qiu et al found overexpression of mRNA and protein of both receptors as well as nuclear colocalization along with upregulation of PCNA, suggesting a mitogenic effect [26]. Other studies have shown, however an inverse correlation of ERβ with Ki67, a marker of cell proliferation [36].

Some clinical and in vitro studies suggest that imbalanced ERα/ERβ expression is a common feature and could be a critical step in estrogen-dependent tumor progression [37]. ERβ seems to play a key role in the mitogenic action of estrogen, by providing protection against ERα-induced hyper-proliferation. Moreover, a number of molecular mechanisms may explain the differential roles of ERα and ERβ, including telomerase activity, differences in ligand affinity and transactivation, distinct cofactor interactions, and putative heterodimerization. Splicing variant ER isoforms also may be important in modulating the cellular response [38]. The clinical value of ERβ in cancer prognosis and its possible usefulness as a predictor of hormone response should be assessed in large-scale, prospective clinical studies.

In view of our findings we could conjecture that telomerase activity could be modulated by estrogen receptors, which also might regulate the expression of bcl-2. The identification of hTERT as a target of estrogens is a novel finding which advances the understanding of telomerase regulation in hormone-dependent cells, potentially implicating hormones in senescence and malignant conversion [39].

Little is known about how telomerase levels predict responses to treatment in breast cancer. However, Takahashi et al. [40] investigated the relationship between telomerase activity and response to chemotherapy in epithelial ovarian cancer patients in vivo, and reported no significant differences between responders and non-responders. In esophageal cancer, cell lines with high telomerase activity were more sensitive to cisplatin [41,42]. In all prior studies, telomerase activity was assayed by TRAP. The different results obtained may be ascribed, at least in part, to technical problems related to TRAP methodology. In particular, Buttitta [43] considers that one of the main problems in the TRAP assay is that the presence of telomerase inhibitors in the tissue extract may block the enzyme during the TRAP assay, which might also be a problem in the Trapeze method. The method used in this study exhibits considerable sensibility and specificity in the quantitative evaluation of telomerase activity, and establishes different levels of activity. A possible explanation for the technical problems that could have led to false negative telomerase test results is the size of the tissue sample obtained by means of a tru-cut biopsy. The ratio of malignant cells to benign cellular elements was not quantified, but it was clear that >50% of tissue was malignant during histopathological examination.

Our study reveals a link between low levels of telomerase activity and the absence of ERβ expression. Recent studies, in vitro, suggest a common step in estrogen-dependent tumor progression and telomere loss [20]. We found lower telomerase activity in tumors that did not express ERβ. Buttitta et al. [43] investigating the correlation between telomerase activity and treatment response in ovarian cancer discovered that major telomerase activity predicted a better response to chemotherapy. Previous studies have demonstrated that higher telomerase activity correlates with ERα expression [20]. We have not, however, found previous studies of telomerase activity in relation to the expression of ERβ in breast cancer. This study underscores the fact that in ERβ negative tumors telomerase activity is significantly reduced or abolished. These findings may help to elucidate the mechanisms of hormonal control of telomerase activity in estrogen-induced carcinogenesis [20].

## Conclusion

We identified differences in the frequency of the expression of estrogen receptors. Estrogen receptor beta was positive in only 36% of cases while estrogen receptor alpha was found in 88%. Our study revealed a correlation between low levels of telomerase activity and the absence of ERβ expression. These findings may help to elucidate the mechanisms of hormonal control of telomerase activity, and to clarify the respective roles of estrogen-receptor alpha and beta in breast carcinogenesis.

## Abbreviations

hTERT human telomerase reverse transcriptase

ERα Estrogen receptor alpha

ERβ Estrogen receptor beta

TRAP Telomeric repeat amplification protocol

TPG Total Product Generated

DCIS Ductal carcinoma *in situ*

CR Complete Clinical Response

PR Partial Response

NR No Response

## Competing interests

The authors declare that they no have conflict of interest in the elaboration of this investigation.

## Authors' contributions

BM was in charge of selecting the patients, obtaining biopsies, recruiting clinical data, follow-up information and analysis of biomolecular data, also contributed in interpretation of findings and preparing the first draft of the manuscript. HA carried out the TRAP method for quantization of telomerase in tissue extracts. SC was responsible of the microscopic immunocytochemical assessment. JM performed statistical analysis, and helped with the interpretation of findings and elaboration of the manuscript. LB conceived the study, supervised the technical procedures, interpreted data, polished and edited the manuscript. All authors reviewed and accepted the final version of this manuscript.

## Pre-publication history

The pre-publication history for this paper can be accessed here:



## Figures and Tables

**Table 1:** Clinical-pathological data of 44 breast cancer patients.

**Clinical Parameter**	**Median (Range)**	**n**	**(%)**
Age (years)	52 (27–77)		
**Menopausal status**			
Premenopausal		21	(47.7)
Postmenopausal		23	(52.3)
**Clinical Stage**			
II		14	(31.8)
III/IV		30	(68.2)
**Histologic Grade**			
Low		12	(27.3)
Intermediate and High		32	(72.7)
**Tumoral Size**			
< 5 cm		21	(47.7)
> 5 cm		23	(52.3)
**Axillary node status**			
Positive		25	(56.8)
Negative		15	(34.1)
**Treatment Response**			
Complete Response (CR)		29	(65.9)
Partial Response (PR) or No Response (NR)		15	(34.1)
**Telomerase activity**			
Positive		26	(59)
Negative		18	(41)
**Estrogen Receptor α**			
Positive		39	(88)
Negative		5	(12)
**Estrogen Receptor β**			
Positive		16	(36)
Negative		28	(64)
**Bcl-2**			
Positive		35	(79.5)
Negative		9	(20.4)

**Table 2:** Percentage of cellular immunoreactivity of the ERα, ERβ and Bcl-2 and Telomerase Activity.

**Case**	**Bcl-2%**	**ERα%**	**ERβ%**	**Telomerase Activity (TPG Unit)**
1	94	62	0	1.37
2	78	63	0	49.93
3	100	10	0	1.57
4	0	13	0	4.42
5	48	10	0	0.35
6	0	99	98	1.45
7	100	10	98	17.07
8	70	100	90	3.53
9	100	94	100	0
10	27	0	0	0
11	0	60	0	34.7
12	0	65	89	7
13	0	10	0	17.56
14	0	10	100	0
15	100	80	60	11.96
16	98	10	0	25.47
17	100	97	71	4.75
18	93	95	100	5.24
19	0	71	0	0
20	97	50	0	19.29
21	76	78	0	11.75
22	68	65	85	25.78
23	90	100	0	41.26
24	87	91	0	7.89
25	40	100	0	0
26	89	78	10	0
27	89	99	98	0
28	85	98	40	0
29	93	0	0	0
30	94	73	0	0
31	100	90	83	0
32	10	97	73	13.65
33	99	80	0	0
34	56	89	0	4.86
35	88	75	60	0
36	0	0	0	0
37	51	10	0	7.33
38	60	9	0	12.17
39	98	10	0	13.91
40	0	0	0	0
41	25	0	0	0
42	54	98	0	2.98
43	55	100	0	0
44	63	10	0	0

**Table 3:** Correlation between molecular biomarkers and histological grade in all breast cancer specimens.

**Histological grade**	**Low Grade**	**Intermediate and High Grade**	***X^2^***	***P value***
	**n (%)**	**n (%)**		
**Telomerase activity**				
Positive	8 (40)	18(75)	4.5	0.033*
Negative	12 (60)	6(25)		
**Bcl-2**				
Positive	10(83)	25(78)	0.14	0.7
Negative	2(17)	7(22)		
**ER alpha**				
Positive	12(100)	27(84)	2.11	0.15
Negative	0 (0)	5 (16)		
**ER beta**				
Positive	4(32)	12(37)	0.65	0.8
Negative	8(68)	20(63)		

*Fisher's exact test (two-tailed). p significance at ≤ 0.05.

**Table 4:** Correlation between clinical stage and tumor biomarkers.

	**STAGE II (%)**	**STAGE III/IV (%)**	***X^2^***	***P Value***
**Telomerase activity**				
Positive	7 (50.0)	19 (63.3)	0.7	0.4
Negative	7 (50.0)	11 (36.7)		
**Bcl-2**				
Positive	14(100)	21 (70)	5.28	0.02*
Negative	0 (0)	9 (30)		
**Estrogen Receptor α**				
Positive	13(92.9)	26 (86.7)	0.36	0.55
Negative	1 (7.1)	4 (13.3)		
**Estrogen Receptor β**				
Positive	4 (28.6)	12 (40)	0.54	0.46
Negative	10(71.4)	18 (60)		

*Fisher's exact test (two-tailed). p significance at ≤ 0.05.
